# Method for Measurement of Multi-Degrees-of-Freedom Motion Parameters Based on Polydimethylsiloxane Cross-Coupling Diffraction Gratings

**DOI:** 10.1186/s11671-017-2289-0

**Published:** 2017-08-30

**Authors:** Junping Duan, Qiang Zhu, Kun Qian, Hao Guo, Binzhen Zhang

**Affiliations:** Key Laboratory of Instrumentation Science and Dynamic Measurement, North University of China, Taiyuan, 030051 China

**Keywords:** Cross coupling diffraction gratings, Multi-degrees of freedom, Motion parameter measurement, PDMS

## Abstract

This work presents a multi-degrees-of-freedom motion parameter measurement method based on the use of cross-coupling diffraction gratings that were prepared on the two sides of a polydimethylsiloxane (PDMS) substrate using oxygen plasma processing technology. The laser beam that travels pass the cross-coupling optical grating would be diffracted into a two-dimensional spot array. The displacement and the gap size of the spot-array were functions of the movement of the laser source, as explained by the Fraunhofer diffraction effect. A 480 × 640 pixel charge-coupled device (CCD) was used to acquire images of the two-dimensional spot-array in real time. A proposed algorithm was then used to obtain the motion parameters. Using this method and the CCD described above, the resolutions of the displacement and the deflection angle were 0.18 μm and 0.0075 rad, respectively. Additionally, a CCD with a higher pixel count could improve the resolutions of the displacement and the deflection angle to sub-nanometer and micro-radian scales, respectively. Finally, the dynamic positions of hovering rotorcraft have been tracked and checked using the proposed method, which can be used to correct the craft’s position and provide a method for aircraft stabilization in the sky.

## Background

Multi-degrees-of-freedom motion parameters can provide accurate location and attitude information about a specific target, which have been widely used for large structures in applications such as the control of aircraft attitude stability, the aiming stability of gun probe systems, robotic arm movement, the alignment of precision parts, and workpiece positioning for industrial processing [1–3].

Therefore, high-precision detection methods were used to sense multi-degrees-of-freedom information (e.g., straightness, pitch, and deflection angle) about targets, and these methods required high-performance sensors, including the characteristic of high-speed detection, synchronization, high measurement precision, and in real time. These methods were widely used in aerospace, unmanned aerial vehicle, precision manufacturing, and optical alignment applications [4–6].

The method of precise real-time measurement and decoupling of the dynamic multi-degrees-of-freedom movement information were the key elements to determine the carrier attitude stability. Hsieh [7] proposed a three-dimensional detection array that used three groups of modules for sensing the different degrees of freedom, in which different sensing modules were used to measure the different position information and an algorithm was used to calculate the angular and multi-degrees-of-freedom information. Liu [8] presented a multi-degrees-of-freedom motion parameter measurement method based on change in the relative angle between two assembly gratings to perform the information measurements. However, the above approach was prone to be the errors from assembled two or more sensing elements and the complexity of the coupling calculations, and its accuracy also depends on the high-precision instrument system.

With the development of micro-nano manufacturing technology, nanotechnology, and nanomaterials, researchers have studied the multi-degrees-of-freedom motion parameter detection methods based on the single-chip implementation, from the perspective of miniaturization and low-cost application in the fields of nanomaterials, optical materials, and nanodevices. Tana [9] reported a multi-degrees-of-freedom movement parameter detection algorithm with a non-diffracting beam based on a portable miniaturized prism structure, which could minimize the measurement errors. Our team has presented a vectorial strain gauge method based on a single sensing element which can be applied to surface vectorial strain measurements using multi-axis integrated mechanical sensors and provided the foundation for the research in this paper [8, 10].

In this work, a multi-degrees-of-freedom vector displacement and angle measurement method have been demonstrated based on a single element; this element was fabricated using oxygen plasma processing technology to form an orthogonal gradient optical grating structure on both sides of a polydimethylsiloxane (PDMS) substrate that was pre-bent into an ellipse shape. These crossed optical gratings can cause an input laser beam to be diffracted into a two-dimensional spot array. The diffraction spot location information can be used to achieve the incident beam angle calculated by the location algorithm in real time. Based on this method and a 480 × 640 pixel charge-coupled device (CCD), the measurement resolutions of the displacement and the deflection angle were 0.18 μm and 0.0075 rad, respectively. Additionally, higher pixels CCD can improve the measurement resolution of the displacement and the deflection angle to sub-nanometer and micro-radian levels, respectively. Finally, the dynamic position of a hovering rotorcraft has been tracked using the proposed method in real time; the information acquired can be used to correct the craft’s position and it proves a new method for aircraft stabilization in the sky.

## Experimental

### Polydimethylsiloxane (PDMS) Preparation

The PDMS (Sylgard 184) was purchased from Dow Corning. PDMS (10:1) membranes were prepared by spin coating on silicon wafers and were cured immediately after spinning at temperatures of less than 80 °C for 2 h. PDMS substrates with a thickness of 600 μm were prepared by controlling the spinning speed.

### Double Orthogonal Grating Preparation

**Fig. 1:**
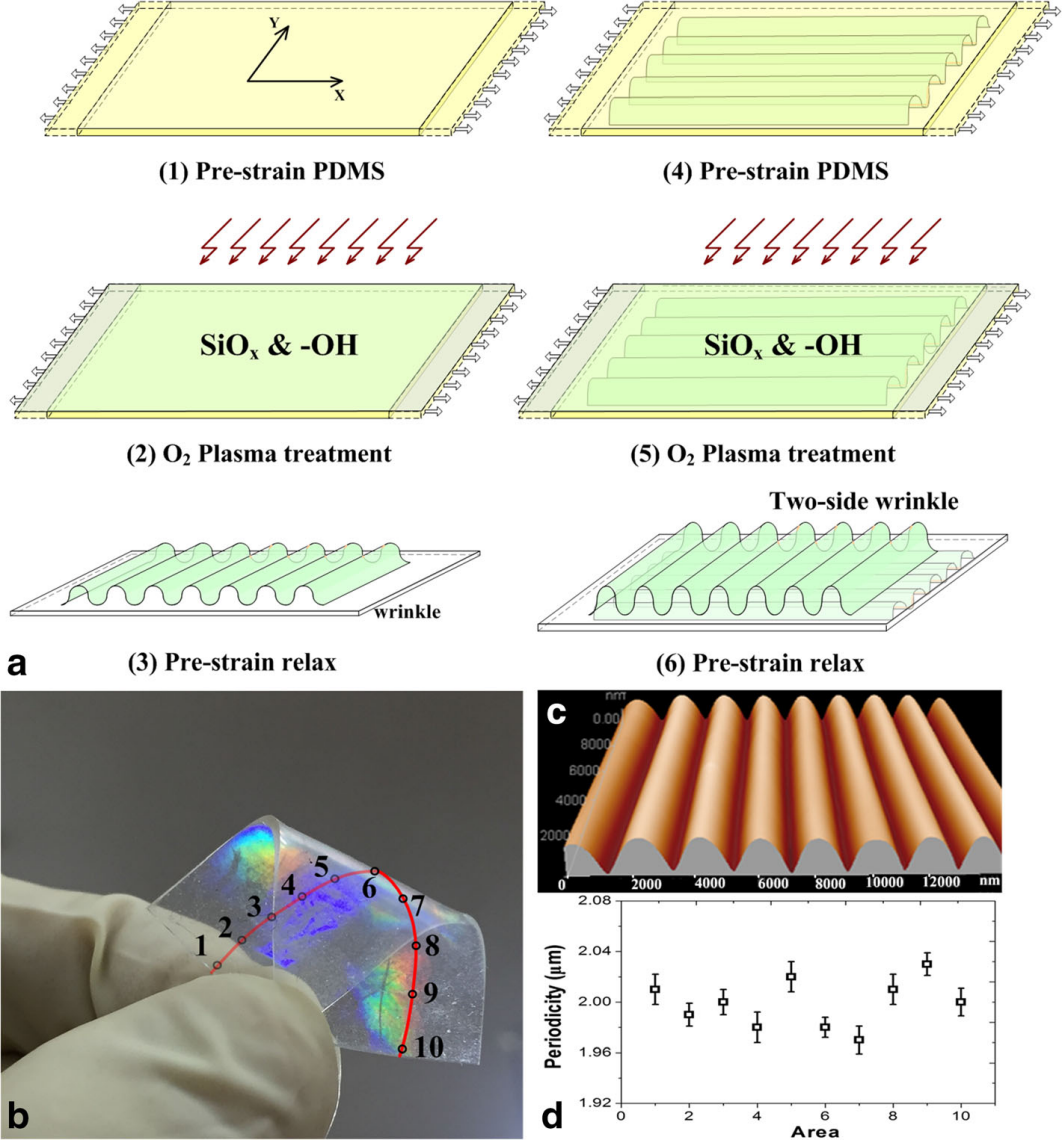
Fabrication process and morphology characterizations of PDMS double optical grating. **a** Fabricating double optical grating. **b** The optical images of grating. **c** Atomic force microscopy image of the grating. **d** The uniformity of periodicity for the samples

According to the experimental requirements, the PDMS films were prepared with an area of 3 × 3 cm^2^. The PDMS films were then pre-strained relative to the original by 1.5 times in the X direction using a homemade translation stage. Wrinkled SiO_*x*_ layers were then formed on the O_2_ plasma-treated pre-strained PDMS substrate (IoN Wave 10, PVA-TePla, Germany) under conditions of an oxygen flow rate of 30 sccm and an oxidation time of 40 s. An even and orderly nanograting structures were formed on the surface of PDMS substrate after relaxed the pre-strained. As shown in Fig. 1a, this process was repeated taken on the other side of the PDMS substrate with an angular difference of 90°, to form the orthogonal grating structures on both sides of the PDMS substrate.

### Test Platform Building

**Fig. 2:**
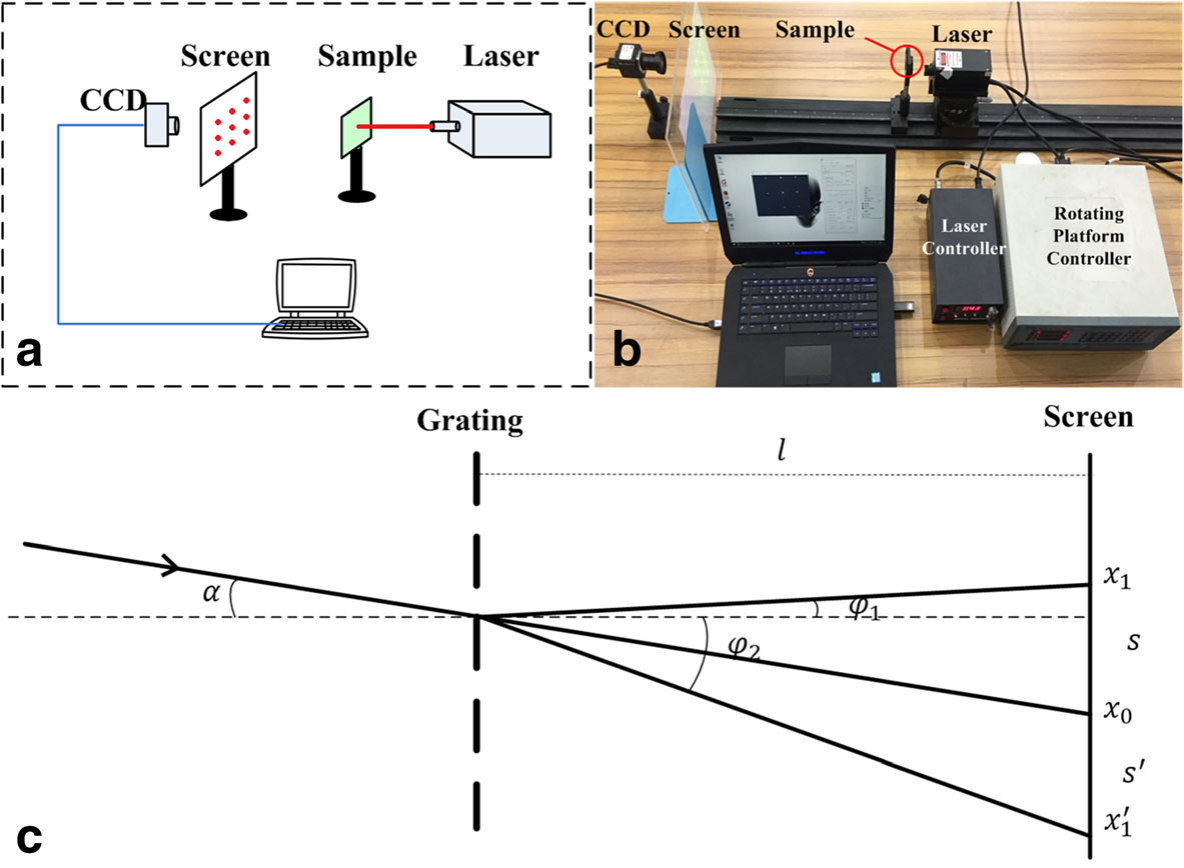
The principle and test system for the MODF motion parameter. **a** system diagram. **b** System setup. **c** Testing principle of displacement and angle

The four-degrees-of-freedom displacement angle sensor system have been built includes a laser light source, an angle and displacement platform assembly, a specimen holder, a screen, a CCD camera, and a computer. As shown in Fig. 2a He-Ne laser light source (laser wavelength 680 nm) was installed in the angle and displacement platform assembly that consisted of an electric rotating platform and a manual three-dimensional adjustment frame (Beijing Zolix Instrument Co., Ltd.). The platform has the rotational accuracy of 0.1° and the displacement precision of 2 μm. This crossed optical grating can cause the laser beam to be diffracted into a two-dimensional spot array. A complementary metal-oxide-semiconductor (CMOS) camera with 480 × 640 pixels was used to acquire an image of the two-dimensional spot array in real time using MATLAB image processing algorithms, which was used to extract each diffraction point location and then calculate the *x-* and *y*-axis displacements and the angle information. A test platform in the form of a four-rotor aircraft (Typhoon Q500, Yuneec Electric Aviation) was provided. And four-degrees-of-freedom information was acquired to obtain the indoor hovering attitude.

## Analysis and Discussion

### Orthogonal PDMS Grating Characterization

The fabrication process was shown as in Fig. 1a. Hydrophilic surface modification of PDMS used the oxygen plasma technology. A SiO_*x*_ layer and hydrophilic groups (e.g., −OH) were thus formed on the pre-bent PDMS substrates by the oxygen plasma. When the pre-strain in the PDMS substrate exceeds a critical value, grating structures were formed on the surface of PDMS after pre-strain relaxing [11, 12]. The periodicity of the gratings was achieved by tuning of the applied pre-bending and plasma conditions and can be calculated in our previous works. As shown in Fig. 1c, the topographies of the micro-/nanogratings were characterized by atomic force microscopy (AFM) (CSPM5500; Benyuan Co.). As shown in Fig. 1b, d, 10 areas have been selected along the center line on the one side of the sample to study the periodicity and the uniformity of grating structures. The corresponding periodicity of gratings of 10 areas was uniform and had a period of (2 ± 0.05) μm over the whole sample surface.

### Diffraction Grating for Position and Angle Motion Parameter Characterization

The laser beam travels pass the sample (with the grating) to diffract into light spots matrix, according to the of Fraunhofer diffraction theory [13]. The diffraction spot position was directly related to the position and the angle of the incident beam, and thus the position information of the incident beam can be detected by the location information of diffraction spots.

Figure 2 shows the moving and rotating platform to track the positioning and the corresponding diffraction spots of incident beam. According to Fraunhofer diffraction theory, when the diffraction grating and the screen distance are fixed, then the relationship between the incident beam, the diffracted beam, and the wavelength can be expressed as follows:

1 $$ d\left(\sin \varphi \pm \sin \alpha \right)= m\lambda \left(m=0,1,2,\dots \right) $$

Here, *λ* was the wavelength of the incident beam, *d* was the period of the grating, *α* was the incidence angle, *φ* was the diffraction angle, and *m* was the grating diffraction order.

When the angle of incidence *α* was not equal to 0, “+” then indicates that the diffraction beam and the incident beam distributed on the same side of the grating normal, while “–” indicates that the diffraction beam and the incident beam exist on two sides of the normal. At a specific angle of incidence, the distances between the first order of diffraction points and the zero order of diffraction points were not equal on the screen. Therefore, the distance between the points can change with the angle of incidence. The angle of the incident beam can be calculated quantitatively through calculation of the diffraction light spot position. Simultaneously, the mobile location of the incident beam causes movement of the zero order of diffraction point. The incident beam position information can be calculated by the location information of the zero order of diffraction beam point.

Figure 2c shows a single direction of the grating diffraction diagram, where *x*_0_ was the first order of diffraction spots, and *x*_1_ and ${x}_1^{\hbox{'}}$ indicate the second order of diffraction spots. From the Fig. 2c, *s* and *s*’ were the distance between the first and second order of diffraction spots which were expressed as follows:

2 $$ s=l\tan \alpha +l\tan {\varphi}_1 $$

3 $$ {s}^{\hbox{'}}=l\tan \alpha -l\tan {\varphi}_2 $$

From the Eq. (1):

4 $$ d\left(\sin {\varphi}_1+\sin \alpha \right)=\lambda $$

5 $$ d\left(\sin {\varphi}_2-\sin \alpha \right)=\lambda $$

From the above, the correlation model between beam incident angle and diffraction speckle spacing can be obtained as:

6 $$ s=l\tan \alpha +\tan \left(\arcsin \left(\frac{\lambda }{d}-\sin \alpha \right)\right) $$

7 $$ {s}^{\hbox{'}}=l\tan \alpha -\tan \left(\arcsin \left(\frac{\lambda }{d}+\sin \alpha \right)\right) $$

### Orthogonal Diffraction Grating-Based Multi-Degrees-of-Freedom Motion Parameter Detection and Characterization

**Fig. 3:**
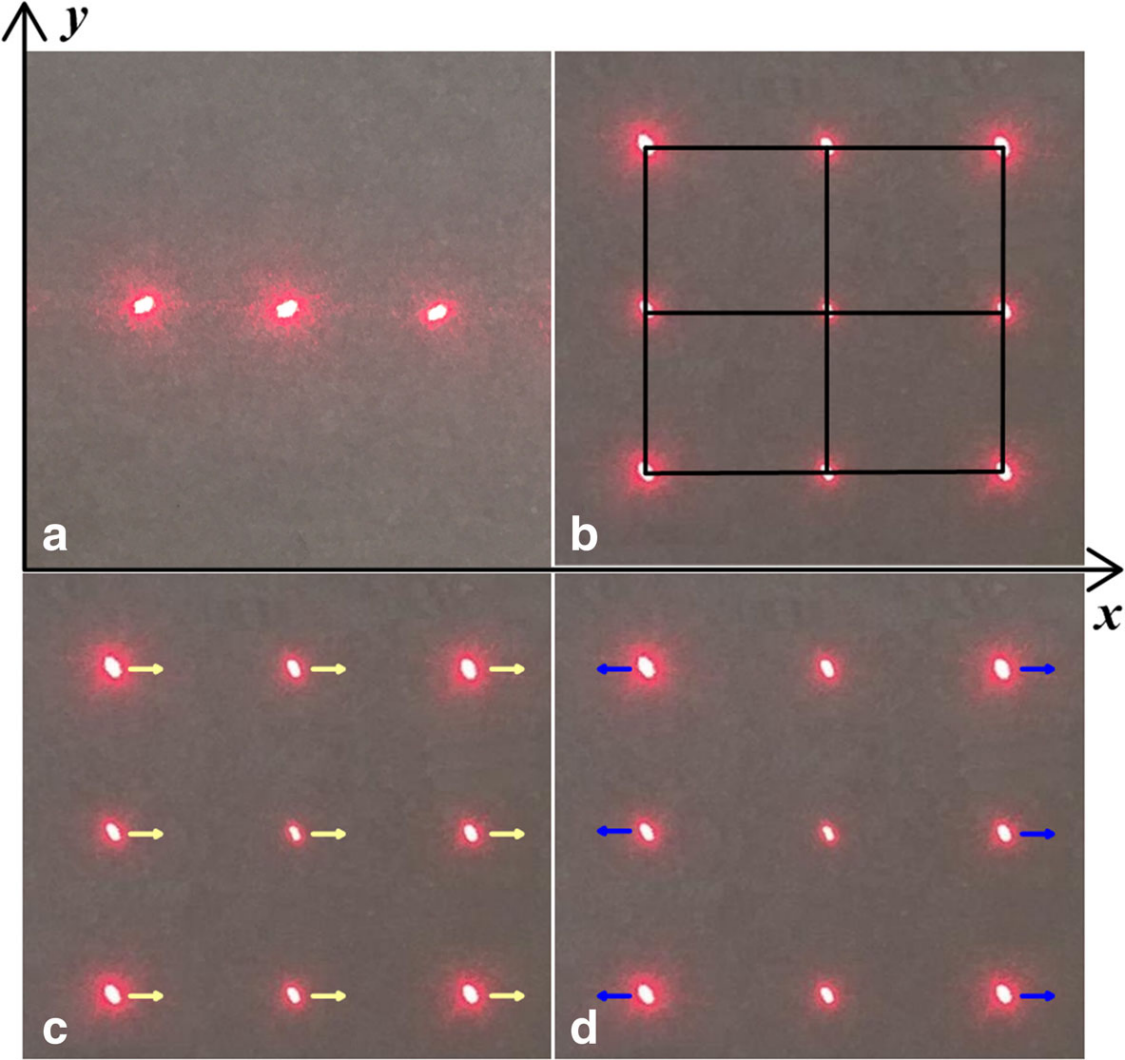
The MODF motion parameter depends on the moving of diffraction spots. **a** One-dimensional diffraction spots were generated by the single-direction grating. **b** Two-dimensional spot array was generated by the double cross optical grating. **c** Moving of spot array was controlled by the moving of laser source. **d** Gap moving between the spot array was controlled as the incident angle of laser beam

Laser beam that travels pass a single-direction optical grating can form the single diffraction spots. The orthogonally oriented can be formed as the laser beam travels pass the orthogonal gratings on the two sides of PDMS substrate. A one-dimensional grating diffraction beam will be formed when a light beam is transmitted along the direction of the grating on one side on the screen and the dimensions were set in the *x*-axis. A one-dimensional grating diffraction beam was then formed orthogonal to the *x*-axis when a light beam passes along the direction of the grating on the other side on the screen and the dimension was then set in the *y*-axis. A two-dimensional diffraction point array was formed on the screen, as shown in Fig. 3b.

When the position of a laser beam was changed, the zero order of diffraction light spot position will show a corresponding movement, and the position of the diffraction bitmap will change accordingly based on Fraunhofer diffraction theory. The incident beam position can be directly calculated based on the direction of lattice movement, and then detected the realize position information of light beam along the *x-* and *y*-axis. As shown in Fig. 3c, the first order of the diffraction point position cannot accurately calculate the light displacement due to the coupling effects of the displacement and the deflection. Additionally, the zero order of diffraction point location was only related to the source location. Therefore, it would be more accurate to use the zero order of diffraction point displacement to calculate the position of light source. As shown in Fig. 3d, the deflection angle information of the incident light beams along the *x*-axis and the *y*-axis can be calculated by the distance between of light spot on the *x*-axis and the *y*-axis based on the related model between the angle and the change of the spot.

However, limitation to the reason of the displacement of diffraction spots depends on the incidence angle and the distance between the grating and the screen based on the Eq. (1). In our works, the grating was fixed with the screen, which means the distance variation between the grating and the screen was zero. There was non-displacement of diffraction spots when the laser source was moving along the *z*-axis. As well, when the laser source was rotating along the *z*-axis, the incidence angle variation was zero, which would result to non-displacement of diffraction spots.

In our experiments, the angle change (Δ*θ*_*x*_) along the *x*-axis can be calculated in terms of the column spacings (*s*_*x*_,${s}_x^{\hbox{'}}$) of the diffraction spots, and the angle change (∆*θ*_*y*_) along the *y*-axis can be calculated based on the column spacings (*s*_*y*_,${s}_y^{\prime }$) of the diffraction spots. The portfolio platform was adjusted to change the light source location, and then the camera images were acquired by MATLAB software every 0.02 s to extract the diffraction spots position for comparison with earlier values, which was used to calculate the spot array displacements on the *x*-axis and the *y*-axis and the changes in the column spacing and row spacing of the array.

Based on the algorithm, the displacement of spot array can be analyzed by handling image before and after the movement to calculate the ∆*x*,∆*y*, ∆*θ*_*x*_, and ∆*θ*_*y*_. Because the laser spot includes multiple pixels in the image and its energy was according with the Gaussian distribution, the Gaussian distribution fitting method was used to remove the background noise from the image to extract the laser spot center location accurately. The Gaussian function of the laser spot is expressed as follows:

8 $$ I\left(x,y\right)=H\cdot \exp \left\{-\left[\frac{{\left(x\hbox{-} xo\right)}^2}{\sigma_1^2}+\frac{{\left(y\hbox{-} yo\right)}^2}{\sigma_2^2}\right]\right\} $$

Here, *I* (*x*, *y*) was the spot intensity and *H* was the amplitude, (*x*_0_, *y*_0_) was the light spot center coordinates, and *σ*_1_, *σ*_2_ were the standard deviations on the *x*-axis and the *y*-axis, respectively.

A logarithm can be applied to both sides of the above equation to obtain the spot center location, which can be expressed as follows:

9 $$ {x}_0=-\frac{c}{2a} $$

10 $$ {y}_0=-\frac{d}{2b} $$

Here, *a*, *b*, *c*, and *d* were the polynomial coefficients that were obtained by Gaussian fitting of all pixels in the spot.

The changes of distance between two diffraction spots have been calculated by two images before and after the movement. And the center spot of diffraction spots has been set as the system of coordinate centers before the movement: the absolute displacement and the relative displacement coordinate systems of the light spot. The absolute displacement coordinate system of the diffraction light spot took a stillness screen as a reference. The movement information (*Δx*,*Δy*) of the lattice in both screens can be calculated by the zero order of diffraction point coordinate (i.e., the center position). The relative displacement coordinate system for the light spot took the zero order of diffraction spot as a reference, which can be used to calculate the changes in the spot-array spacing (*S*_*x*_) and the row spacing (*S*_*y*_).

**Fig. 4:**
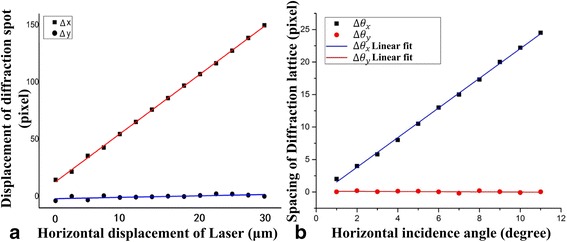
Characterization of four degrees of freedom. **a** Displacement of laser source depends on the displacement of the diffraction spots. **b** Incident angle of laser source depends on the gap between of diffraction spots

Figure 4 showed the characterization of the four degrees of freedom. When the laser beam was mowing along the *x*-axis, there was a corresponding movement of diffraction lattice in the *x*-axis, but the displacement was about zero in the *y*-axis. The sensitivity of displacement was about 5.4 pixels/μm. This method can be used to calculate the location information for the light source along the axis with the high accuracy, as shown in Fig. 4a.

When the laser rotated a small angle along the *x*-axis, there was a corresponding distance change of diffraction spot-array row spacing and the spot-array column spacing was zero. The sensitivity of displacement was about 2.3 pixels per angle (/°). Meanwhile, the measuring range of angle was about 9.8° in theory calculated by the Eqs. (1)–(5) as the distance *s* = 0. Attributing to first order of diffraction spots coincides with the zero order of diffraction spots as increasing the incident angle, the distance change of diffraction spots would be zero (*s* = 0). This method can be used to achieve the angle information of the light source along the *x*-axis. The location and angle information can also be obtained using this method.

The detection resolution of one pixel depend on the algorithm based on the MATLAB software. As calculated above, the method has a displacement sensitivity of 5.4 pixels/μm, which means the resolution was 0.18 μm*.* For the displacement sensitivity of 2.3 pixels*/°*, it was a resolution of 0.0075 rad. This shows that, based on the method presented here and the CCD resolution, the resolutions of the displacement and angle were 0.18 μm and 0.0075 rad, respectively. The 480 × 640 pixel CCD was used to acquire the image of the two-dimensional spot array in real time. Additionally, higher pixels CCD and optimization of the light path could improve the resolutions of the displacement and the deflection angle up to sub-nanometer and micro-radian scales, respectively.

### Hovering Aircraft Rotor Motion Parameter Information Characterization

A rotorcraft was a type of civil unmanned aircraft system with low precision, which was widely used in the aerial, model aircraft, and navigation fields. Stability control of a rotorcraft represents a microcosm of an unmanned combat platform. To realize high-precision flight control, the most important aspect was steady control of the aircraft’s attitude and position. And the core aspect was decoding of the high-precision hovering attitude and position information in real time, such that the accurate four-degrees-of-freedom motion parameter information about hovering becomes an essential asset.

In our experimental, based on a cross-coupled diffraction grating, a measurement method has been presented to achieve the four-degrees-of-freedom attitude information of aircraft flight in real time. First, a four-rotor aircraft was used to replace the platform, which was composed of the position and posture of a four-degrees-of-freedom test system, which was based on a double grating to set up the four-degrees-of-freedom attitude testing system for four-rotor aircraft. In the test system, a small laser pointer was fixed at the center of a four-rotor aircraft as the light source and it shines the laser beams vertically downwards. A sample with the double grating, a screen, and a camera turn along the center of the optical axis. This crossed optical grating can cause the laser beam to be diffracted into a two-dimensional spot array. In the experiments, the camera was used to acquire the picture from the screen and transmit images to computer in real time to calculate the displacement information by the MATLAB software.

**Fig. 5:**
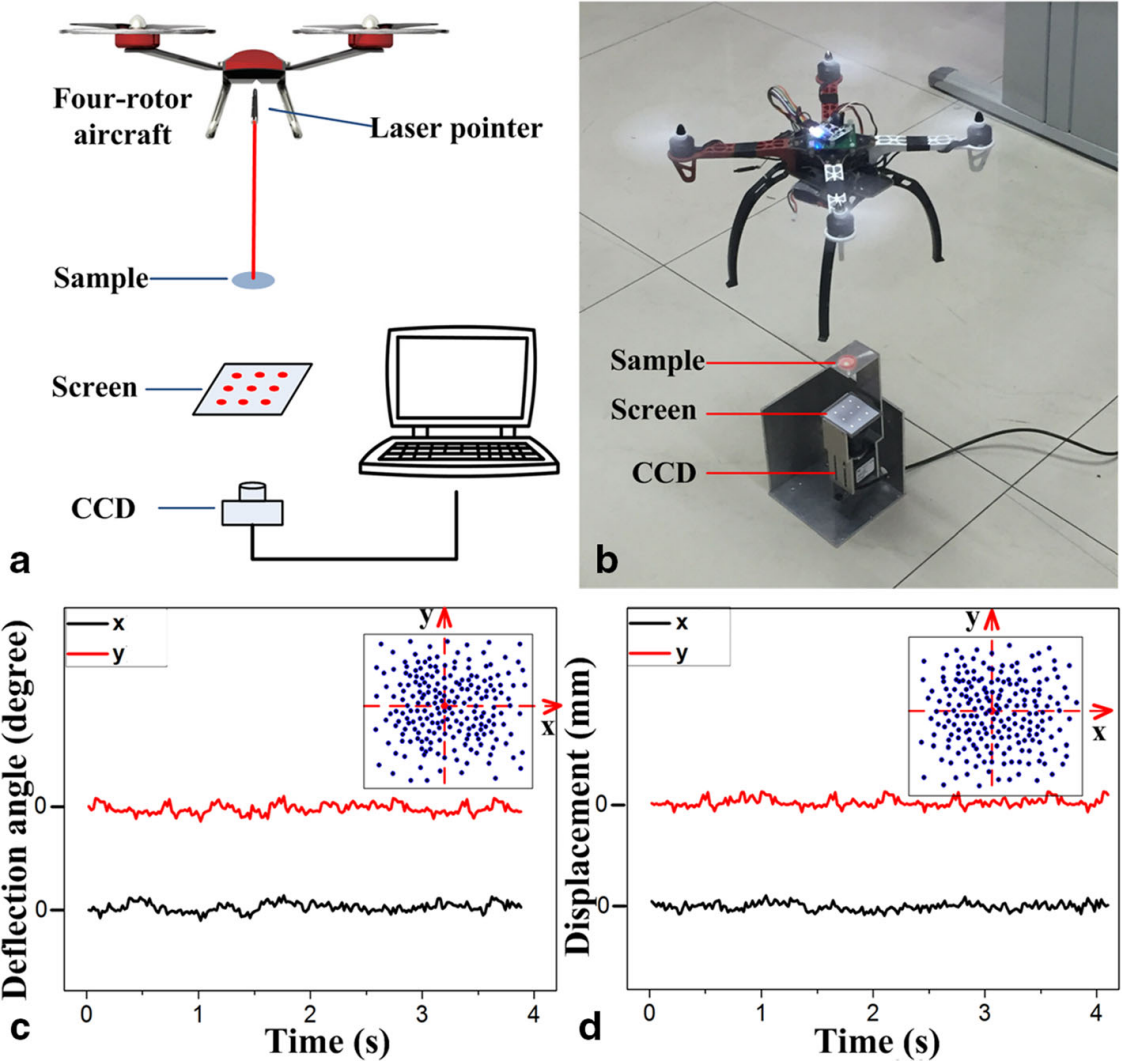
Characterizations of four-rotor craft attitude. **a** System diagram. **b** Setup of system. **c** Angle of deflection of rotor craft. **d** Displacement of rotor craft

To achieve rapid, accurate, and real-time measurements of the flying signals, four-rotor aircraft was hovering in the air and fast tracking the posture signal maintained for 4 s. Information about the axial displacements along the *x*-axis and the *y*-axis for four-rotor aircraft have been obtained in time of 4 s, as shown in Fig. 5c. On the basis of establishment of a planar coordinate system (i.e., an *x*-axis and a *y*-axis), the *x* and *y* values are converted into these coordinate points. The 200 positioning results in 4 s mean one point acquired in 0.02 s. This represents use of the tracking method for the aircraft every 0.02 s in real time to determine its location and position. The aircraft has a maximal displacement of 2.1 mm in the *x*-axis and maximum displacement of 2.3 mm in the *y*-axis, according to the algorithm.

Additionally, the pitch angle and roll angle information for the aircraft have been calculated by the above algorithm and data processing method. As shown in the inset of Fig. 5d, the rotation angle dot diagram of the four-rotor aircraft delivered accurate angle information for aircraft by tracking in real time every 0.02 s. It observed that the aircraft has a maximum angular deviation of 1° in the *x*-axis and the *y-*axis. This method can thus calculate the four-degrees-of-freedom information for aircraft, which can feedback the accurate position and angle signals to the flight control system within 0.02 s to improve the stability of the aircraft.

## Conclusions

In summary, a simple manufacturable technology was demonstrated to fabricate the orthogonal optical grating structure with periodicity of 2 μm on the two sides of the PDMS substrate. Based on the orthogonal optical grating structure, a method has been studied to identify the beam position and an angular motion parameter information using the diffraction light spot position information based on the Fraunhofer diffraction effect. A 480 × 640 pixel CCD was used to acquire images of the two-dimensional spot array in real time. The results show that, when using this method and the CCD described above, the resolutions of the displacement and the deflection angle were 0.18 μm and 0.0075 rad, respectively. Additionally, with the higher pixel CCD, the resolutions of the displacement and the deflection angle can improve up to sub-nanometer and micro-radian scales, respectively. This method can be used to detect accurate hover positions and angle information for rotor aircraft in real time with high accuracy every 0.02 s. The information can give back to control the flight system for unmanned aerial vehicles in the air. This method was simple, low-cost, and high precision and can realize real-time monitoring while providing a research foundation for stable flight and precise control of aircraft for unmanned combat platforms.
